# Supplementary figures and images for: Electromyographic measures of asymmetric muscle control of swallowing in Parkinson’s disease
Source: PLoS One. 2022 Feb 18;17(2):e0262424. doi: 10.1371/journal.pone.0262424 (PMC8856551; doi:10.1371/journal.pone.0262424)

| 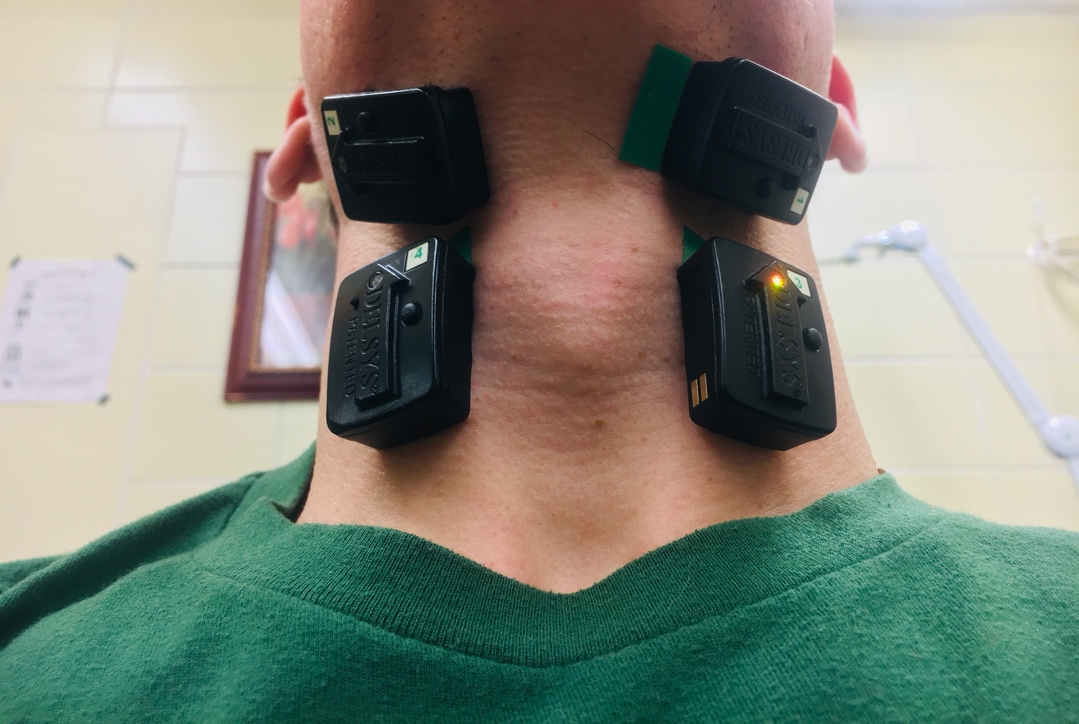S1 Fig. Data collection set up. |
| --- |

Supplement: S1 Fig — (DOCX) [file pone.0262424.s001.docx]
