# Supplementary material for: Electromyographic measures of asymmetric muscle control of swallowing in Parkinson’s disease
Source: PLoS One. 2022 Feb 18;17(2):e0262424. doi: 10.1371/journal.pone.0262424 (PMC8856551; doi:10.1371/journal.pone.0262424)
Supplement: S1 Table — (DOCX) [file pone.0262424.s002.docx]

S1 Table**.** Statistical results for differences between the more and less affected Side.

| Most Affected Vs. Least Affected Side (mean) | Swallow Conditions | Z Score | P-Value |
| --- | --- | --- | --- |
| Laryngeal Rise Time | THICK | -.547 | 0.584 |
| Laryngeal Rise Time | THIN | -.122 | 0.903 |
| Laryngeal Fall time | THICK | -1.673 | 0.09 |
| Laryngeal Fall time | THIN | -.791 | 0.429 |
| Submental Rise Time | THICK | -.639 | 0.523 |
| Submental Rise Time | THIN | -1.065 | 0.287 |
| Submental Fall time | THICK | -.730 | 0.465 |
| Submental Fall time | THIN | -.456 | 0.648 |
